# In the post-COVID-19 era, is the illegal wildlife trade the most serious form of trafficking?

**DOI:** 10.1186/s40163-021-00154-9

**Published:** 2021-09-13

**Authors:** J. Sean Doody, Joan A. Reid, Klejdis Bilali, Jennifer Diaz, Nichole Mattheus

**Affiliations:** 1 Department of Integrative Biology, University of South Florida-St. Petersburg Campus, 140 7th Ave. South, St. Petersburg, FL 33705 USA; 2 Department of Criminology, University of South Florida-St. Petersburg Campus, 140 7th Ave. South, St. Petersburg, FL 33705 USA

**Keywords:** Wildlife trafficking, COVID-19, Cost of crime, Policy

## Abstract

Despite the immense impact of wildlife trafficking, comparisons of the profits, costs, and seriousness of crime consistently rank wildlife trafficking lower relative to human trafficking, drug trafficking and weapons trafficking. Using the published literature and current events, we make the case, when properly viewed within the context of COVID-19 and other zoonotic diseases transmitted from wildlife, that wildlife trafficking is the most costly and perhaps the most serious form of trafficking. Our synthesis should raise awareness of the seriousness of wildlife trafficking for humans, thereby inducing strategic policy decisions that boost criminal justice initiatives and resources to combat wildlife trafficking.

## Introduction

Despite its apparent seriousness, wildlife trafficking ranks low in comparison to trafficking of humans, drugs and weapons when considering the relative crime costs, profits and seriousness (May, 2017). The source of complacency and the tepid societal response to the problem of wildlife trafficking may be rooted in societal perceptions of the harms linked to wildlife trafficking in comparison to other more notorious forms of trafficking (South & Wyatt, 2011; Wagner et al., 2019). Herein we make the case that wildlife trafficking, when properly viewed within the context of COVID-19 and other zoonotic diseases transmitted from wildlife (e.g., SARS from civets), is the costliest and perhaps most serious form of trafficking, despite the seriousness of trafficking in humans, drugs, and weapons. Given that seven new coronaviruses have jumped from animals to humans since the 1960s (Cui et al., 2019), the likelihood is very high for future zoonotic epidemics and pandemics if ‘atypical’ human-wildlife contacts continue (Li et al., 2020). We discuss the lack of awareness and underestimation of the wildlife trafficking problem and the complexity of how to tackle trafficking, including pragmatic issues and cultural sensitivities.

### Biodiversity, overexploitation and wildlife trafficking

Biodiversity loss is one of the most severe human-caused global environmental problems (Ceballos et al., 2017). Extinction has always been a feature of life on Earth, but the human domination of global ecosystems has caused a sharp rise in the rate of extinctions to far above pre-human levels (Barnosky et al., 2011; Johnson et al., 2017). Hundreds of species are going extinct annually, and queueing up behind them are immeasurable shrinking populations as we enter the earth’s sixth mass extinction (Barnosky et al., 2011; Ceballos et al., 2017; Dirzo et al., 2014; Sanchez-Bayo and Wyckhuys 2019; Wagner, 2020; Young et al., 2016). Not only are we losing our natural heritage at an alarming rate, humans are reliant on biodiversity, and so by stripping the earth of its living resources we risk human suffering and catastrophe (Daily, 1997; Naeem et al., 2012). For example, biodiversity provides humans with clean air and water; food; mitigation from floods and droughts; detoxification and decomposition of wastes; soil generation, renewal and fertility; pollination of crops; pest control; medicines; climate control; and protection from climate extremes. Despite this dire situation, public awareness of the biodiversity crisis is alarmingly low (Ceballos et al., 2017).

The major global drivers of biodiversity loss are habitat loss and overexploitation, in that order, ahead of invasive species, climate change and pollution (Hoffmann et al., 2010; Maxwell et al., 2016; Ripple et al., 2017). Overexploitation, also known as overharvest, is typically defined as unsustainable exploitation of animals, plants and other organisms. The sale or exchange by people of these resources, overexploited or not, is the ‘wildlife trade’, which can range from local subsistence through small-scale income to large profit-oriented business. Wildlife is traded locally, nationally, regionally or internationally (WCS and Traffic 2004; Blundell & Mascia, 2005; Schlaepfer et al., 2005; Nijman & Shepherd, 2007). Some wildlife trading is legal and some illegal, with the latter referred to as wildlife trafficking, poaching, or wildlife crime. The proportion of the wildlife trading that is illicit is unknown but expected to be large; we are unsure because the very nature of wildlife trafficking is such that reliable data are difficult to obtain (Broad et al., 2003). There are related estimates: seized wildlife products and parts (~ 6 million) was 0.7% of magnitude of the legal trade (~ 900 million) of wildlife exported to the USA during 1979–2014 (Olsen et al., 2021). Uses of trafficked wildlife include traditional medicine, food, apparel, furnishings, pets, gardens and manufacturing (Broad et al., 2003). Wildlife trafficking occurs for a number of reasons including profit, exchange, subsistence, personal ownership, cultural or religious beliefs, or as a consequence of human-animal conflict (McFann & Pires, 2018).

Despite the lack of a comprehensive review, the effects of wildlife trafficking and overexploitation are abundant and widespread. In a sample of 362 species of large vertebrates (> 40 kg), Ripple et al., (2019) found that 59% are threatened with extinction and 70% are decreasing; the top (IUCN Red List) threat for each class was human consumption for meat or body parts, which was also a threat for 98% of all species. Freshwater megafauna (mainly fish > 30 kg in mass) populations for which there were sufficient data (N = 126 spp.) exhibited even larger declines (88%) over the last 40 years, with almost half exhibiting marked range contractions (He et al., 2019). In a meta-analysis of hunting trends across the world’s tropics, Benítez-López et al., (2017) found marked reduction in abundance of mammals (83%) and birds (58%) in hunted areas compared to areas without hunting. Overexploitation was the leading threat to ~ 8700 species of threatened or near-threatened species when logging was included (Maxwell et al., 2016) and overfishing has depleted marine life in similar ways (Halpern et al., 2008; Jackson, 2008; Jackson et al., 2001). Overexploitation is threatening iconic species such as whales, elephants, rhinoceros, and gorillas with extinction, but is also the principal threat to some less-conspicuous groups of animals (e.g., turtles and seahorses; Stanford et al., 2020; Vincent et al., 2011). The surge in demand for animals for Asian traditional medicine is exerting heavy tolls on wildlife and threaten many species with extinction (Ellis, 2013). For example, the exploitation of Asian bears for bile, which is used as a treatment for illness (Feng et al., 2009), is the leading cause of their decline (Fredriksson et al., 2008; Garshelis et al., 2008), and 101 species of primates are killed for traditional medicine and magic-religious rituals throughout the world, of which 64 are conservation-listed (Alves et al., 2010).

## Wildlife trafficking and COVID-19

At the time of writing this paper, COVID-19, the human illness borne out of the novel coronavirus, SARS-CoV-2 (Zhou et al., 2020), has become a pandemic that has infected more than 128 million people and killed more than 2.8 million worldwide, with cases and deaths still on the rise (Worldometer Coronavirus Cases, 2021). Mental health issues and suicide are expected to increase during the pandemic (Gunnell et al., 2020; Lee, 2020), and many survivors are expected to face chronic health problems due to COVID-19. Direct medical (financial) costs in the U.S. alone will be in the hundreds of millions of dollars over the course of the pandemic (Bartsch et al., 2020). The world economic cost of COVID-19 is already in the trillions of dollars (Jones et al., 2020), and the world is currently in the worst recession since the Great Depression, based on the magnitude of negative GDP growth (Gopinath, 2020).

**Fig. 1:**
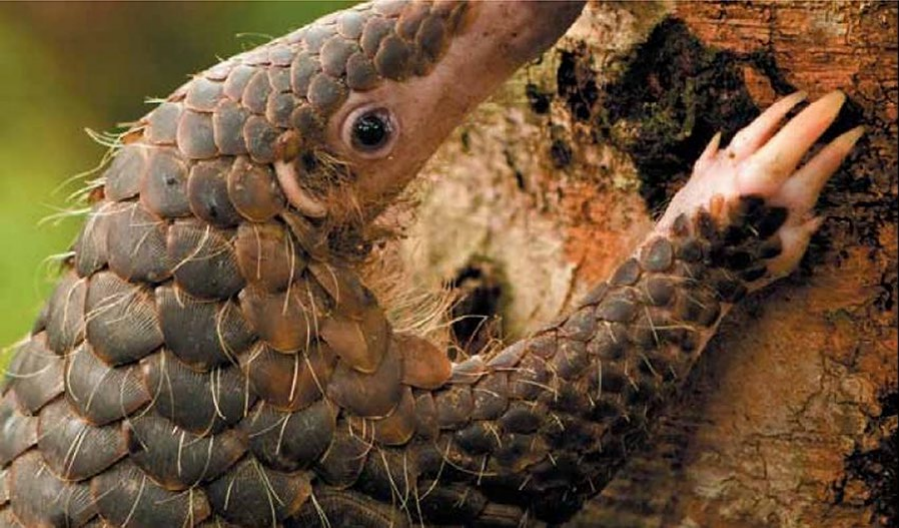
Malayan or Sunda Pangolin, *Manis javanicus*

The current consensus is that the novel coronavirus that causes COVID-19 (SARS CoV-2) ‘jumped’ from its likely natural reservoir in bats into humans via Malayan pangolins (*Manis javanica*, Fig. 1) in a wet market associated with most of the first human COVID-19 cases, in Wuhan, China (Huang et al., 2020; Li et al., 2020). Research revealed that Pangolin-CoV is 91% identical to both SARS-CoV-2 and to Bat-CoV RaTG13 (Zhang et al., 2020) at the whole-genome level, and a pangolin intermediate was corroborated by other research using genomics, amino acids and proteins (Lam et al., 2020; Lopes et al., 2020; Xiao et al., 2020).

Commercial trade in wild-caught Malayan pangolins has been illegal since 2000, and all species of pangolins are now CITES listed (Challender et al., 2015). Malayan and other species of pangolins are highly sought after in China and Vietnam, where their meat is considered a delicacy, and their scales, blood and other body parts are used for traditional medicine to allegedly cure diseases and increase wealth (Zhou et al., 2014; Challender, 2011; Challender et al., 2015; Cheng et al., 2017). Thus, Malayan pangolins bearing the most recent ancestor of the novel coronavirus were illegally trafficked, inexorably linking COVID-19 with wildlife trafficking, forcing a reassessment of the costs of wildlife trafficking.

### Illegal trafficking: pre-COVID-19 comparisons

When considering the comparative rankings of trafficking crimes regardless of whether they are ranked by illegal profits, economic and social costs, or seriousness, wildlife trafficking is consistently ranked lower than drug trafficking and human trafficking, and less serious than weapons trafficking (e.g., May, 2017; Fell et al., 2019; Forte et al., 2017). As we will argue, these rankings are important because they reflect the underestimation of the relative costs of wildlife trafficking for society. When using crime rankings of these forms of trafficking to impress upon society the gravity of these problems, crimes are commonly ranked by illegal profits, by economic and social costs, and by seriousness (see Table 1).

**Table 1:** Comparison of pre-COVID-19 report rankings of trafficking crimes by profits, costs, and seriousness

Report emphasis	Wildlife trafficking	Drug trafficking	Weapons trafficking	Human trafficking
Illegal profits (May, 2017)	3	1	4	2
Economic and social costs (Fell et al., 2019)	N/A	1	3	2
Seriousness (Forte et al., 2017)	4	1	3	2

Ranking from 1 to 4 with 1 being the highest ranked

#### Trafficking crimes ranked by illegal profits

When ranking the various forms of trafficking by estimated annual profit, wildlife trafficking ($5–$23 billion) is ranked behind trafficking in drugs ($426–$652 billion) and persons ($150.2 billion) (May, 2017, see also, Haken, 2011; Lautensach and Lautensach, 2020; Warchol, 2004). Only weapon trafficking ($1.7–$3.5 billion) is ranked lower than wildlife trafficking in terms of profits (May, 2017; see also Clark 2020; Lautensach and Lautensach, 2020).

#### Trafficking crimes ranked by economic and social costs

Economic and social costs of crimes vary by country (Pūraitė, 2020). Crime impacts on the economy are measured by the expenditures for or damage to state and public security including defensive expenditures, costs related to law enforcement and judicial system, publicly-funded legal defense costs, and costs to the prison and probation services. Measures of crime costs also include ecological damage, property stolen, emotional and physical impact and reduced quality of life for victims, reduced labor effectiveness for those impacted by crime, costs to human health and welfare, economics, business order and state finances (Pūraitė, 2020). For example, human trafficking impacts communities by enabling the spread of HIV and other infectious diseases (Kloer, 2010) and threatens public security by generating enormous profits for terrorists, armed groups and criminal organizations (Okubo and Shelley 2011). In the case of drug trafficking, this crime may generate such a huge amount of illegal profits that its prominence deters investment and impairs the capacity of governments to promote sustainable economic growth (Van Dijk, 2007). Illegal wildlife trafficking, by removing wildlife, forest products and coastal resources, results in the loss of ecosystem services such as carbon storage, water filtration and flood retention with estimated annual cost of a staggering $2–$3 trillion (World Bank, 2020). Moreover, losses due to wildlife trafficking threaten benefits to humans (e.g., finding new medicines) and their livelihoods (e.g., local cultures and their economies). Most studies on the comparative costs of crime investigate the costs of street crime such as homicide, assault, sexual assault, burglary, and exclude costs of trafficking or organized crimes (Chalfin, 2015). One of the few comparative reports of economic and social costs of organized crime in the United Kingdom (UK) estimated that drug trafficking incurred annual costs to the UK of ₤20 billion, human trafficking incurred annual costs ₤2.3 billion, and costs of ₤190 million related to weapons trafficking (Fell et al., 2019). Wildlife trafficking was mentioned but the authors did not have the necessary data available to calculate an estimation of the economic and social costs. It is important to note that the single report covered here by Fell and colleagues (2019) is based on the organized crime costs to the UK, a developed demand-side country, and the effects of wildlife trafficking are more direct and larger for source countries of wildlife.

#### Trafficking crimes ranked by seriousness

Lastly, crime is often ranked by seriousness. Crime seriousness is most commonly grounded in public perception of the level of harm of the crime to persons and society as well as the wrongfulness of the crime (Wagner et al., 2019). In a study of perceptions of the seriousness of wildlife crime, researchers found that wildlife crime ranked as less serious, less wrong, and less harmful than personal crimes and property crimes (Wagner et al., 2019). Crime seriousness informs the allocation of resources and establishment of policy priorities and spending related to crime prevention and crime control (Adriaenssen et al., 2018). For example, a 2017 European Union (EU) study categorized the level of threat from serious and organized crimes. This study categorized drug trafficking, human trafficking, and online weapon trafficking as “priority crime threats” and “high threats” to the EU economy, while wildlife trafficking was categorized lower than the other types of trafficking. Wildlife trafficking was categorized as a “threat” but not a “high threat” or “priority crime threat” (Forte et al., 2017). The report of threat priorities do not simply inform the public but are used to direct EU priorities in the fight against serious and organized crime for next EU Policy Cycle from 2018 to 2021 (Forte et al., 2017).

When considering the comparative rankings of trafficking crimes regardless of whether they are ranked by illegal profits, economic and social costs, and seriousness, wildlife trafficking is consistently ranked lower than drug trafficking and human trafficking, and less serious than weapons trafficking (see Table 1). A better understanding of the costs and seriousness of wildlife trafficking is needed to better inform policy development. Without reliable data on the costs of wildlife trafficking, policymakers are unable to craft meaningful policies, and this can lead to erroneous conclusions about the efficacy of proposed policies. As will be discussed in the next section, the COVID-19 pandemic has exposed the world to the reality of the overwhelming costs and seriousness of wildlife trafficking.

### Origin of COVID-19 (SARS-CoV-2): the link to wildlife trafficking

The current pandemic COVID-19, which stands for coronavirus disease 2019, is caused by the novel virus SARS-CoV-2. SARS-CoV-2 is in the beta coronavirus group and is most commonly found in bats (Banerjee et al., 2019; Hampton, 2005; Li et al., 2005; Zhou et al., 2020). For example, SARS-CoV-2 shares 96% of its whole-genome identity with a bat coronavirus called BatCoV RaTG13 found in the intermediate horseshoe bat (*Rhinolophus affinis*) from the Yunnan Province, China (Zhou et al., 2020). Despite its parsimonious link with bat coronaviruses, SARS-CoV-2 is likely to have made its way into a human host via an intermediate species because coronaviruses typically (e.g., SARS CoV and MERS) pass into intermediate hosts before leaping into humans (Cui et al., 2019). The virus in humans is thought to have originated at the Huanan Seafood Wholesale Market in Wuhan, China; 27 of the first 41 patients diagnosed with COVID-19 were linked to the market (Huang et al., 2020; Li et al., 2020). The market closed on 1 January 2020, making it difficult to identify the intermediate vector; the market sold live, wild mammals, but not bats (Wong et al., 2020).

In October, 2019, around the time when COVID-19 was first reported, researchers using metagenomics detected a ‘new SARS-CoV-2-like’ coronavirus (named Pangolin-CoV) in two dead Malayan pangolins (*Manis javanica*), or scaly anteaters (Fig. 1), seized in China, that exhibited a frothy liquid in their lungs and pulmonary fibrosis (Liu et al., 2019). Subsequent research revealed that, at the whole-genome level, Pangolin-CoV is 91% identical to both SARS-CoV-2 and to Bat-CoV RaTG13 (Zhang et al., 2020). Moreover, the S1 protein of Pangolin-CoV was much more closely related to SARS-CoV-2 than to Bat-CoV RaTG13, and five key amino acid residues were 100% consistent with SARS-CoV-2, compared to 4 amino acid mutations in Bat-CoV RaTG13 (Zhang et al., 2020). Metagenomic sequencing of lung, intestine and blood samples from Malayan pangolins identified viral sequences that belong to two sub-lineages of HCoV-19-related coronaviruses, which including five critical residues on the receptor binding domain of the pangolin virus that are identical to SARS-CoV-2 (Lam et al., 2020). Moreover, further characterization of SARS-CoV-2 (Xiao et al., 2020) and a phylogenetic analysis of proteins that could form a species-specific barrier that interferes with bat-human transmission (Lopes et al., 2020) implicates Malayan pangolins as the intermediate host for SARS-CoV-2. Thus, although we are far from certain (Choo et al., 2020; Huang et al., 2020; Wong et al., 2020), the most likely transmission vector of SARS-CoV-2, based on current knowledge, was from bats to pangolins to humans.

The Malayan pangolin is one of eight species of pangolins (order Philodota) worldwide, including four species in southeast Asia and four species in Africa (Gaudin et al., 2009). Pangolins are medium-sized, mainly solitary, nocturnal mammals that feed almost exclusively on ants and termites (Macdonald et al., 2004). All eight species are highly sought after in China and Vietnam, where their meat is considered a delicacy, and their scales, blood and other body parts are used for traditional medicine to allegedly cure diseases and increase wealth (Challender, 2011; Challender et al., 2015; Zhou et al., 2014), despite no reliable evidence of the medicinal efficacy of their scales or other body parts (Cheng et al., 2017).

**Fig. 2:**
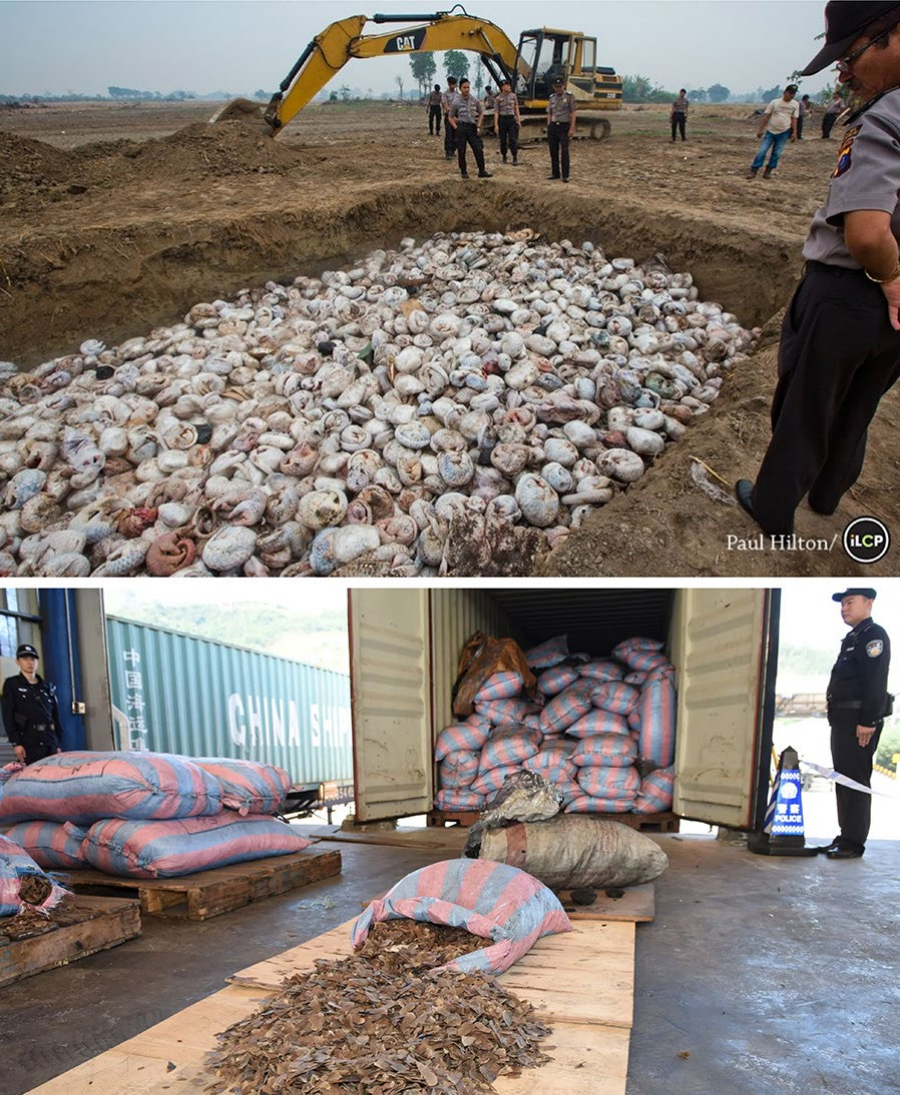
Trafficked pangolins seized by customs officials. Top: thousands of slaughtered pangolins await burning in a pit after being seized by Indonesia National Police and Wildlife Conservation Society’s Wildlife Crimes Unit (29 April, 2015). Photograph by Paul Hilton. Bottom: Chinese customs officials seize 13.1 tons of pangolin scales from up to 30,000 individual pangolins at the port of Shenzhen (29 November, 2017). Photograph by Echo Huang

These apparent benefits have caused significant overexploitation in pangolins over the last few decades (Fig. 2) (Chaber et al., 2010; Challender & Hywood, 2012; Challender et al., 2020; Chin & Pantel, 2009; D’Cruze et al., 2018; Harrington et al., 2018; Heinrich et al., 2016; Katuwal et al., 2017; Mohapatra et al., 2015; Nijman et al., 2016; Zhang et al., 2017). Pangolins are vulnerable to overexploitation due to their low population densities and low rates or reproduction (Harrington et al., 2018; Mahmood et al., 2014, 2015; Pietersen et al., 2014; Zhang et al., 2016). Commercial trade in wild-caught Malayan pangolins has been illegal since 2000, and all species of pangolins are now CITES listed (Challender et al., 2015). The Malayan pangolin is listed as Critically Endangered, while the other seven species have listings ranging from Vulnerable (the four African species) to Threatened or Endangered (the other three Asian species) (ICUN 2020). Despite these conservation listings, it is estimated that > 895,000 pangolins were illegally trafficked globally during 2000–2019 (Challender et al., 2020). Pangolins, as a result, are considered to be “the world’s most trafficked mammals” and “an icon of the illegal wildlife trade” (Aisher, 2016; Harrington et al., 2018). Trafficking from both African and Asian pangolins is predominately destined for China and Vietnam (Challender et al., 2020), and the illicit trade remains the key threat to survival in all pangolin species, despite impacts of local hunting and domestic use (Baillie et al., 2014; Challender et al., 2014b, c, Pietersen et al., 2014; Waterman et al., 2014; Challender et al., 2020).

SARS-CoV-2 is one of a multitude of human emerging infectious disease (EID) with its origin in non-human animals (zoonosis). The coronaviruses causing SARS (SARS-CoV) and MERS (MERS CoV), which killed ~ 700 and ~ 800 people and infected ~ 8000 and ~ 2500 people, respectively (Stadler et al., 2003; Zumla et al., 2015; Shehata et al., 2016; de Wit et al., 2016), originated from bats (the natural reservoir), but passed through the intermediate hosts of civets and camels, respectively (Guan et al., 2003; Zaki et al., 2012; Ge et al., 2013; Azhar et al., 2014; Kupferschmidt, 2014). Taylor et al., (2011) catalogued 1415 known human pathogens, of which 62% had zoonotic origin. Although domestic animals can be reservoirs, most zoonoses originate in wildlife (Allen et al., 2017; Greger, 2007; Karesh et al., 2012; Kruse et al., 2004; Wolfe et al., 2007). Notorious examples include HIV, Ebola, Rabies, West Nile, Malaria, bubonic plague, swine flu, bird flu, salmonella, anthrax, and typhus.

The increased exposure of humans to various trafficked wildlife species increases risks of new EIDs. For example, the myriad of unique combinations of illegally harvested wildlife in wet markets can promote host-jumping in potentially deadly pathogens. Moreover, while zoonosis related to local consumption in rural or remote areas can allow containment—for example, the Ebola virus in central Africa, trafficking wildlife into areas with dense human populations poses increased risks of uncontainable spread as seen in SARS-CoV-2. Wildlife trafficking thus brings a unique and potentially deadly vector for zoonosis. Given that there have been seven new coronaviruses to infect humans over the last ~ 50 years (Cui et al., 2019), it highly likely that there are more to come (Li et al., 2020).

To summarize, in the future, more new zoonotic viruses can be expected to jump into humans from bats or other animals via intermediate hosts, some of which will cause serious disease. Even if pangolins are not the intermediate host for COVID-19, they could very well transmit their strain of the virus (Pangolin CoV) to humans, as could other wild animals sold in wet markets. For example, animals that are susceptible to COVID-19 and thus carriers include cats, hamsters, ferrets, monkeys and tigers (reviewed in Wong et al., 2020). The odds of such zoonosis are greatly increased by human-animal interactions associated with wildlife trafficking (Li et al., 2020), given the unique combinations of animals and contact with humans in markets and probably along trafficking routes. Accordingly, the trend of increasing zoonotic virus emergence is expected to continue (WHO/FAO/OIE 2004). As early as 2003, the Institute of Medicine report on emerging infections suggested that without appropriate policies and actions, the future could bring a “catastrophic storm of microbial threats” (Smolinski et al., 2003). Of six major risk factors identified as driving emerging zoonoses (WHO/FAO/OIE 2004), wildlife trafficking includes four: the increasing demand for animal protein, long-distance live animal transport, live animal markets, and bushmeat consumption.

### COVID-19 as collateral damage of wildlife trafficking

The COVID-19 pandemic has infected more than 184 million people and killed more than 4 million, worldwide (Worldometer Coronavirus Cases, 2021). At the time of writing, 6–9 thousand people are dying every day, worldwide (Worldometer Coronavirus Cases, 2021). Although daily death rates have leveled or are declining in many countries, they are increasing steadily in some countries such as Russia, Indonesia and Bangladesh (Worldometer Coronavirus Cases, 2021). Mental health issues and suicide are expected to increase during the pandemic (Gunnell et al., 2020; Lee, 2020); for example, an additional 10,000 persons committed suicide after the 2007–2008 global financial crisis (Reeves et al., 2014).

The world macroeconomic cost of COVID-19 is already in the trillions of dollars (Jones et al., 2020), and the world is currently in the worst recession since the Great Depression, based on the magnitude of negative GDP growth (Gopinath, 2020). For instance, the total cost of COVID-19 for the U.S., presuming its decline in Autumn 2021, has been estimated at 16 trillion dollars (Cutler & Summers, 2020). Direct medical (financial) costs in the U.S. alone will be in the hundreds of billions of dollars over the course of the pandemic (Bartsch et al., 2020; Hackett 2020). There are also microeconomic costs. For example, about 50% of ~ 10,000 survey participants reported household income and wealth losses, of which the averages were $5,293 and $33,482, respectively (Coibion 2020). Moreover, the costs associated with the COVID-19 pandemic are not just about the losses of lives and direct financial burden, but include opportunity costs (Ataguba, 2020). For example, removal of the ability to work will put a strain on families (Bonnet et al., 2019), and the pandemic may reduce funding for other critical health priorities such as communicable, nutritional and infectious diseases (Ataguba, 2020).

In comparison to the costs of the COVID-19 pandemic summarized above, the costs of trafficking of humans, drugs and weapons are relatively lower when using several available estimates. If costs are measured in human lives, COVID-19 deaths (4 million) overshadow the estimated 750,000 deaths linked directly or indirectly to illicit drug use (Global Burden of Disease Study, 2017) and the estimated 245,000 lives lost due to illegal firearms annually (INTERPOL 2017). However, all of these estimates are limited—there is no global data on indirect deaths—those deaths caused by disease and the lack of food, clean water and health care that result from wars facilitated by illegal weapons trafficking. Less is known about deaths related to human trafficking—with estimates as low as 2 homicides per year in Europe related to human trafficking (Walby et al., 2020). Other figures suggest, the lives lost due to human trafficking are much higher. For example, since 2014, the International Organization for Migration (IOM) Missing Migrants Project has recorded the deaths of more than 32,000 people globally—these fatalities must include victims of human trafficking (Singleton, 2019). However, this number is likely a gross underestimate because the majority of migrant deaths around the world go unrecorded. Nevertheless, the number of lives lost due to trafficking of humans, drugs and weapons pales in comparison to the loss of life due to COVID-19.

If costs are measured in annual healthcare costs, in the U.S. illegal drug use is estimated to result in $11 billion in direct healthcare costs (National Drug Intelligence Center, 2011) and $3–$6 billion in annual healthcare costs are related to gun violence (Fransdottir & Butts, 2020). Annual healthcare costs for victims of trafficking in the U.S. is not available; however, increased usage of healthcare and social projection by victims of human trafficking in the 27 countries of the European Union (including the UK) is estimated to cost approximately EUR 20,749 per victim annually—extrapolated based on the number of registered (identified and presumed) human trafficking victims to total EUR 245 million annually (Walby et al., 2020). The actual number of human trafficking victims is likely to be significantly higher and the healthcare costs presented here are an underestimate. Even with recognition that these are likely underestimates of the true costs, the healthcare costs related to trafficking of humans, drugs and weapons are substantially lower when compared with the projected estimates of the direct healthcare cost related to COVID-19, which range from $163.4 billion to $546.6 billion in the U.S. (Bartsch et al., 2020; Hackett 2020), and in comparison to the estimated EUR 13.9 billion spent on direct healthcare costs of COVID-19 patients in the EU during the first wave of COVID-19 (January–June 2020) (Czernichow et al., 2021).

Given the gargantuan cost of COVID-19 to society, and the likelihood that wildlife trafficking was the vehicle for zoonosis of the COVID-19-causing pathogen, we contend that, overall, wildlife trafficking may be the most serious and costly form of trafficking (i.e., vs. humans, drugs, weapons). This conclusion is supported by the facilitation by wildlife trafficking of zoonosis for the SARS pathogen. Given the recent emergence of new coronaviruses and other zoonotic diseases (e.g., seven new coronaviruses since the 1960’s, Cui et al., 2019), we can expect future catastrophic, zoonotic pandemics to emerge if wildlife trafficking is allowed to continue to operate as per usual. Thus, a perfunctory or *laissez-faire* treatment of wildlife trafficking by governments will likely bear a future cost that far outweighs tackling wildlife trafficking at the present time.

## Conclusion: awareness and policy changes going forward

Although it is not our intention here to review the myriad ways of addressing and combatting wildlife trafficking, the seriousness of the pandemic serves to refocus our attention on ways forward. Chief among these is raising awareness and education of wildlife trafficking’s damage to biodiversity and human society (Zhang et al., 2008), while at the same time understanding what factors drive the wildlife trade. Although wildlife has been traded illegally and fought for by conservationists for many decades, within criminology there are emerging subfields—“green”, “eco-global”, or “conservation” criminology—with shared concerns regarding environmentally damaging forms of criminality emphasizing that the degradation of the environment is ultimately more harmful to humans than street crime (e.g., Lynch, 1990; Carrabine et al., 2004; Bierne & South, 2007; White, 2009; Sollund 2016; Kurland & Pires, 2017; Sollund, 2019; South & Wyatt, 2011). Green criminology recognizes the role played by whole societies, including individuals, corporations and governments, in draining limited natural resources, and the associated negative consequences for humans, including causing environmental disasters (Lynch, 1990). Traditional conservationists continue to fight wildlife trafficking on the ground—often one species at a time—under the mantra that biodiversity is inherently valuable in its own right. But, biodiversity conservation is value-laden; much of society sees no inherent value for biodiversity beyond its use for, or effects on, humans. By revealing the profound damage wildlife trafficking inflicts upon human society, green criminology can help illuminate the role for a healthy biodiversity for human prosperity.

Recently, permanent bans on “wet markets” based in China have been proposed as a response to COVID-19. Policy makers that fervently endorse these measures have argued that wet markets serve as breeding grounds for wildlife trafficking, providing traffickers with easy access to exotic wildlife. Conversely, some have argued that philosophies behind bans are often rooted in xenophobic perceptions of second- and third-world countries, many of which accommodate diverse populations of people that, for generations, have built their livelihood through such means (Roe et al., 2020; Lee & Houston, 2020). Banning such spaces, they argue, may kindle cultural, economic, and environmental repercussions, with destitute communities bearing the brunt of such drastic decisions (Roe et al., 2020). Equally important to note is the cultural significance that wet markets sustain. Roe and colleagues (2020) explain that these spaces are prized in Chinese culture because of the freshness of the foodstuff. To reduce the demand for species with zoonotic potential, Western powers must first understand that attempts to change these attitudes may be perceived as insolent by Chinese persons because Western individuals are unlikely to recognize the significance of wildlife consumption in established Chinese customs (e.g., the use of pangolin scales for medicinal purposes) (Margulies et al., 2019; Zhu & Zhu, 2020). Demand reduction techniques rooted in Western values are not always culturally nuanced, and therefore, not well-received by consumers of Eastern cultures (Margulies et al., 2019). Motivations to change eating behaviors must be internalized by Chinese individuals and cannot stem strictly from external pressures such as sanctions imposed by international agencies with Western values.

Further, the utilization of celebrities in demand-reduction advertisements has long been perceived as a means of encouraging biodiversity conservation. Yet, awareness on wildlife trafficking should aim to communicate with audiences that anyone can contribute to this global problem; that is, conservation campaigns should refrain from promoting adverts with xenophobic undertones that target specific cultural groups, particularly populations of Asian heritage. Margulies and colleagues (2019) argue that traditional conservation campaigns have painted individuals of Asian backgrounds as unrestrained “super consumers” of exotic wildlife—a crucial point, considering the host of racist attitudes and hate crimes directed toward Asian communities during the span of COVID-19 (Gover et al., 2020). Public awareness campaigns should, then, refrain from pushing forth narratives influenced by Western customs at the expense of Eastern values.

The inclusion of celebrities revered in both, Western and Eastern countries could prove to be valuable in such campaigns. For instance, internationally acclaimed actor Jackie Chan and basketball star Yao Ming have long been involved in public media campaigns (such as those spearheaded by WildAid) that address the overconsumption and illicit trafficking of exotic and endangered animals (Ebiner, 2018; Galster et al., 2010). However, research is somewhat contradictory with regard to use of popular, non-expert socialites as a marketing technique for wildlife conservation. Duthie and colleagues (2017) found that celebrity involvement with conservation campaigns was more likely to attract attention from viewers relative to non-celebrity expert endorsement; however, viewers were more likely to retain marketed information if conservation experts primarily endorsed these campaigns. Additionally, viewers were more likely to engage with campaigns if they perceived that the celebrity was knowledgeable about and genuinely interested in the conservation issue (Duthie et al., 2017). Therefore, celebrity collaborations can be successful, provided that the message is culturally conscious, and therefore, more palatable to audiences of countries engaging in wildlife trafficking, and that celebrity-advocates are known to endorse similar views expressed by the conservation organization.

At the macro-level, policy aiming to combat wildlife trafficking must be strategic. For example, researchers have argued that enacting extreme policies that fail to see much of the ‘gray area’ of wildlife trafficking can drive the trade further underground, given evidence of corruption (Wyatt et al., 2018) and the extent of globalization in the internet age (Lavorgna, 2014; Sollund 2016). More specifically, if a previously legal animal becomes proscribed, but consumer demand remains high, supply chains for that specific animal may not cease, and may indeed increase (Sollund, 2016). Zoonoses can transpire anywhere, with many possible animal carriers (Eskew & Carlson, 2020); therefore, extreme policies must also take into account the possibility that these surreptitious networks may trigger a pandemic that may be difficult to track in origin and predict in transmission. Policy, then, must be strategic and target specific conditions; for example, surveilling large-scale wet markets (Zhu & Zhu, 2020); enforcing strict sanitary regulations that prevent overcrowding within these spaces and requiring adequate conditions for live animals (Roe et al., 2020); abolishing the trade of animals with particularly increased potential for zoonoses (e.g., pangolins and bats) (Aguirre et al., 2020); encouraging sustainable comestible alternatives if possible (Thomas-Walters et al., 2020); and investing in conservation projects aimed at promoting sustainable agriculture to reduce habitat loss among animals with zoonotic potential (Arora & Mishra, 2020; Roe et al., 2020). These policies should not apply solely to second- and third-world countries. Lee and Huston (2020) point out that meat processing plants in North America have facilitated the transmission of COVID-19 among such spaces; thus, policies should not overlook industrialized nations.

Our assertion—that wildlife trafficking has been elevated to the most serious of the big four trafficking types due to causing a pandemic—is our attempt to raise awareness about the seriousness of the effects of wildlife trafficking on society and biodiversity. We have not been comprehensive in our coverage of the effects of wildlife trafficking on biodiversity or on humans—others have accomplished that. In particular, our argument focuses on costs of wildlife trafficking to humans within the context of zoonoses. In crimes directly negatively impacting humans (e.g., human trafficking) the social costs include not only direct financial costs, but also costs associated with victim pain and suffering (Brand & Price, 2000; Cohen et al., 2004). The main victim in wildlife trafficking has been biodiversity; biodiversity suffers as species are harvested and trafficked, driving them towards extirpation and extinction. COVID-19, enabled by wildlife trafficking, has now victimized humans. Doubtless we have also not fully recognized synergistic effects of wildlife trafficking on humans and biodiversity. For example, some organized crime networks involved with wildlife trafficking have also been implicated in both drug and human trafficking (Rademeyer, 2012; Deflem, 2015; South & Wyatt, 2011). Collectively, to be sure, wildlife trafficking is much more serious than public consensus would affirm. It is a multibillion-dollar industry, and its transnational networks are a major contributor to the erosion of biodiversity and present a formidable and serious challenge to conservation. Perhaps a silver lining to the COVID-19 pandemic, through collateral damage in the immense loss of life and innumerable costs to humans, will be a novel recognition of the seriousness of wildlife trafficking to biodiversity and thus, human society.

## Data Availability

Not applicable.
